# Pentraxin 3, ficolin-2 and lectin pathway associated serine protease MASP-3 as early predictors of myocardial infarction - the HUNT2 study

**DOI:** 10.1038/srep43045

**Published:** 2017-02-20

**Authors:** Inga Thorsen Vengen, Tone Bull Enger, Vibeke Videm, Peter Garred

**Affiliations:** 1 Department of Laboratory Medicine, Children’s and Women’s Health, Norwegian University of Science and Technology, Trondheim, Norway; 2 Department of Immunology and Transfusion Medicine, St Olavs University Hospital, Trondheim, Norway; 3 Laboratory of Molecular Medicine, Department of Clinical Immunology, Sect. 7631, Rigshospitalet, Faculty of Health and Medical Sciences, University of Copenhagen, Trondheim, Norway

## Abstract

The lectin complement pathway is suggested to play a role in atherogenesis. Pentraxin-3 (PTX3), ficolin-1, ficolin-2, ficolin-3, MBL/ficolin/collectin-associated serine protease-3 (MASP-3) and MBL/ficolin/collectin-associated protein-1 (MAP-1) are molecules related to activation of the lectin complement pathway. We hypothesized that serum levels of these molecules may be associated with the incidence of myocardial infarction (MI). In a Norwegian population-based cohort (HUNT2) where young to middle-aged relatively healthy Caucasians were followed up for a first-time MI from 1995–1997 through 2008, the 370 youngest MI patients were matched by age (range 29–62 years) and gender to 370 controls. After adjustments for traditional risk factors, the two highest tertiles of PTX3 and the highest tertiles of ficolin-2 and MASP-3 were associated with MI, with odds ratios (95% confidence interval) of 1.65 (1.10–2.47) and 2.79 (1.83–4.24) for PTX3, 1.55 (1.04–2.30) for ficolin-2, and 0.63 (0.043–0.94) for MASP-3. Ficolin-1, ficolin-3 and MAP-1 were not associated with MI. In a multimarker analysis of all associated biomarkers, only PTX3 and MASP-3 remained significant. PTX-3 and MASP-3 enhanced prediction of MI compared to the traditional Framingham risk score alone (AUC increased from 0.64 to 0.68, p = 0.006). These results support the role of complement-dependent inflammation in the pathophysiology of cardiovascular disease.

Atherosclerosis is a leading cause of morbidity and mortality in the industrialized world^1^. The typical clinical manifestations of advanced atherosclerosis are myocardial infarction (MI) and stroke, often happening without any prior clinical warning^2^,^3^. Prediction models based on clinical parameters, such as the Framingham score for calculating the 10-year risk for cardiovascular disease (CVD)^4^, are imprecise and 15–20% of patients admitted with MI have no major traditional risk factors^5^. Thus, early molecular or biochemical warning signals are urgently needed.

Chronic low-grade vascular inflammation plays an essential role in the initiation, progression and destabilization of atherosclerotic plaques^6^. Among the innate immunity components, activation of the complement system has been associated with both pre-lesional stages and the progression of atherosclerotic lesions^7^. However, the mechanisms that drive the persistent non-resolving inflammation in the vessel wall remain incompletely understood.

Activation of innate immunity relies on a set of pattern recognition receptors (PRRs). Recently, long pentraxin-3 (PTX3), a PRR in the same family as C-reactive protein, has emerged as a new candidate risk marker of cardiovascular diseases^8^. Serum PTX3 levels have been associated with disease severity and mortality in patients with acute myocardial infarction^9^, ischemic stroke^10^, cancer^11^, acute respiratory distress syndrome^12^ and sepsis^13^. PTX3 is released by multiple cells including monocytes/macrophages, neutrophils, and activated endothelial cells upon stimulation by IL-1 and TNF-α, cytokines markedly expressed in atherosclerotic lesions. PTX3 recognizes and binds to foreign molecules, leading to activation of the classical and lectin pathways of the complement cascade^14^,^15^.

Several studies have investigated the association between mannose-binding-lectin (MBL), the PRR and initiating factors of the lectin pathway of complement activation, and the risk of MI and cardiovascular disease. Previous results from our group demonstrated that MBL deficiency is associated with an increased risk of severe atherosclerotic disease^16^,^17^, suggesting that there exists a genetic predisposition related to the inflammatory system that makes some individuals more susceptible to coronary artery disease. However, the results from various studies have been conflicting and the role of MBL remains debated^18^.

In addition to MBL, five other PRRs have been reported to be able to activate the lectin pathway by the binding to conserved structures present on various microorganisms or altered host cells: Collectin-10 (collectin liver 1, CL-L1, CL-10), collectin-11 (collectin kidney 1, CL-K1, CL-11), ficolin-1 (M-ficolin), ficolin-2 (L-ficolin) and ficolin-3 (H-ficolin). The PRRs are found in complex with the MBL/ficolin/collectin-associated serine proteases (MASPs), i.e. MASP-1, -2 and -3^19^. In addition, two non-enzymatic proteins associated with the PRRs exists: MBL/ficolin/collectin-associated protein (MAP-1 or Map44) and small MBL-associated protein (sMAP, Map19 or MAP-2). MASP-1 and MASP-2 are crucial for lectin pathway activation, whereas the biological role of MASP-3 remains to be elucidated. Recent evidence indicates that MASP-3 is important for conversion of pro-factor D to active factor D, the initiating enzyme of the alternative pathway of complement, but it may also have a down-regulating effect on the lectin pathway^20^,^21^. sMAP plays a regulatory role in the activation of the lectin pathway^22^, whereas MAP-1 has shown to inhibit the lectin pathway of complement activation^23^.

The complement system is an intricate system consisting of a complicated network of mediators with many interactive effects. We hypothesized that PRRs and their associated proteins in the lectin pathway regulate the immune response in the pathogenesis of atherosclerosis. Thus, the aim of the present study was to investigate the association between serum levels of PTX3, ficolins, MASP-3 and MAP-1 in young and middle aged relatively healthy individuals from the general Norwegian population with the future risk of MI. These might represent novel biomarkers for CVD and potential targets for the development of future treatment strategies.

## Results

### General characteristics investigation

Baseline characteristics of cases and controls are displayed in Table 1. Conventional cardiovascular risk factors were more frequent among cases: They had higher BMI, a more unfavourable lipid profile, and more often hypertension, diabetes, and family history of myocardial infarction. Cases were more often current smokers and had higher Framingham risk scores. Mean age for MI was 53 years (range 29–62).

26 (3.5%) and 10 (1.4%) patients had missing data on smoking and creatinine, respectively. Otherwise, missing values for background characteristics were generally low (<0.2%).

### Correlation between serum biomarkers and conventional clinical variables

In linear regression, the R^2^-values were generally low (<0.1), indicating that the variables included explained a fraction of the plethora of variation in biomarker concentration. PTX3 and MAP-1 were not associated with any risk factors. Ficolin-2 (p < 0.001), ficolin-3 (p = 0.03) and MASP-3 (p = 0.03) were associated with BMI, and ficolin-2 was also associated with smoking (p = 0.01). Ficolin-1 was associated with sex (p = 0.002) and creatinine (p = 0.03).

### Association of serum biomarkers with the risk of future myocardial infarction

Serum concentrations of the biomarkers are displayed in Table 2. PTX3 (p < 0.001) and ficolin-2 (p < 0.001) were higher among cases, also after adjustments for classical risk factors. MASP-3 was lower among cases (p = 0.03) and this remained significant after adjustments for conventional CVD risk factors.

PTX3 was weakly correlated with ficolin-2 (Rho = 0.09, p = 0.02) and MASP-3 (Rho 0.09, p = 0.02). MASP-3 was further correlated with ficolin-1 (Rho −0.1, p = 0.01) and ficolin-3 (Rho −0.17, p < 0.001). MAP-1 was correlated with ficolin-2 (Rho 0.14, p < 0.001).

The two highest tertiles of PTX3 were associated with an increased incidence of MI, also after adjustments for traditional risk factors (Table 3). The two highest tertiles of ficolin-2 were also associated with MI, however after adjustments, only the highest tertile remained significant. The two highest tertiles of MASP-3 were associated with a decreased risk of MI, but in the final model, this association remained only for the highest tertile. When including these three biomarkers in the same analysis, only the two highest tertiles of PTX3 and the highest tertile of MASP-3 remained significant in the analysis adjusted for conventional risk factors (Table 4).

**Figure 1:**
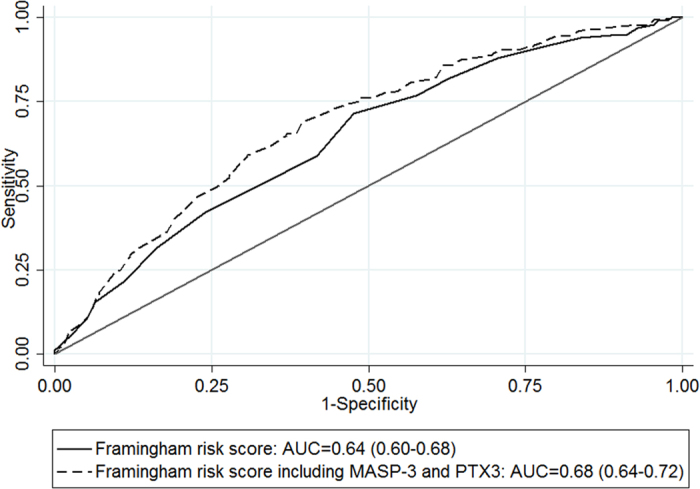
Comparison of the area under the receiver-operating characteristic curve (AUC) of the clinical model based on the Framingham risk score, with the model including pentraxin-3 and MBL/ficolin/collectin-associated serine protease-3.

When adding data on PTX3 and MASP-3 to the traditional Framingham score, the area under the receiver-operating characteristics curve (AUC) was significantly increased from 0.64 (0.60–0.68) to 0.68 (0.64–0.72, p = 0.006, Fig. 1). The continuous net reclassification index showed significant improvement (0.35 (0.21–0.49), p < 0.001). The integrated discrimination index was 0.04 (0.03–0.06), p < 0.001).

## Discussion

The complement system constitutes an important component of innate immunity and atherosclerosis^24^. In the present study, we found that higher concentrations of PTX3 and ficolin-2, and lower concentrations of MASP-3 were associated with increased risk of MI in middle-aged relatively healthy individuals, independent of conventional CVD risk factors.

The associations of higher PTX3 and ficolin-2 levels with an increased risk of future MI may indicate an influence from a higher activity of the complement system. In line with our findings, higher serum PTX3 levels have been related to CVD risk factors, subclinical atherosclerosis and peripheral vascular diseases, as well as clinical CVD events and incident acute coronary syndromes - independently of CRP and CVD risk factors - in older as well as younger, apparently healthy populations^8^,^25^,^26^. PTX3 is rapidly induced at the tissue level and released into the blood at sites of MI, atherosclerosis, vascular damage or inflammatory lesions^27^,^28^,^29^. Thus, PTX3 is increasingly emerging as a potential biomarker of atherosclerosis and CVD^8^. Moreover, PTX3 has also been described as a marker of clinically more advanced cardiovascular disease^30^.

There is increasing evidence that PTX3 possesses atheroprotective properties, suggesting that PTX3 might balance the overactivation of a proinflammatory, proatherogenic cascade^27^. This was further supported by the demonstration that PTX3 deficiency promotes vascular inflammation and atherosclerosis^31^. The available evidence therefore suggests that increased levels of PTX3 in patients with CVD reflects a protective physiologic response, which is correlated to disease severity.

A recent study demonstrated that ficolin-2 and MBL are present in human carotid plaques and recognize cholesterol crystals with subsequent activation of the lectin complement pathway^32^. The present study was not designed to elucidate causal relationships underlying the present associations with MI. However, in previous reports, neither genetic variations influencing blood ficolins nor PTX3 levels influenced the risk of MI^17^,^33^. The missing link between the genetic variants determining PTX3 and ficolin-2 levels with the risk of MI suggests that they act as markers of active atherosclerosis and complement activity rather than causal factors.

When performing a multimarker analysis together with PTX3 and MASP-3 as well as adjusting for conventional risk factors, ficolin-2 was no longer significant. The present correlation matrix showed significant, albeit weak correlation between ficolin-2 and PTX3. Previous reports have shown that ficolins interact with PTX3 and MASPs^15^,^34^. Nevertheless, the lack of association of ficolin-2 to the risk of MI in the multimarker analysis may suggest that the information added by ficolin-2 was at least partially conveyed by the other markers.

Patients suffering acute MI within the follow-up period presented with lower baseline MASP-3 concentrations. Whereas the enzymatic properties of MASP-1 and MASP-2 in the activation of the lectin complement pathway have been described, the role of MASP-3 remains unclear^35^. Low levels of MASP-3 may be related to decreased synthesis or increased turnover. Furthermore, we cannot exclude that MASP-3 acts as a marker for other causal relationships. It has been shown that MASPs interact with ficolins, altering the effect of the complement system. An effect of MASP-3 mediated through interactions with other molecules can therefore not be excluded.

In a recent study investigating the associations of components of the lectin pathway and low-grade inflammation, endothelial dysfunction, carotid intima-media thickness and CVD, MASP-3 was associated with endothelial dysfunction, independent of plasma MBL^36^. The authors suggested a potential role through non-complement pathways. The mechanisms underlying the association between lower MASP-3 concentrations and an increased risk of MI warrants further investigation.

The purpose of this study was not to evaluate the predictive performance of selected biomarkers. Nevertheless, the associations of PTX3, ficolin-2 and MASP-3 may uncover potential new causal mechanisms in the development and progression of coronary heart disease and risk for MI, lead to the generation of new hypotheses and suggest improvements to existing explanatory models. Atherogenesis is characterized by multiple distinct pathways and molecular mechanisms, and our findings underscore the importance of the innate immune response and complement activation. A step further would be to investigate the causal relationships underlying the present associations.

On the other hand, we do not exclude that the described markers may prove useful in a predictive setting. Inclusion of biomarker data significantly improved the AUC, and indicators of predictive ability such as the net reclassification index and integrated discrimination index indicated potential usefulness in clinical prediction. There is a growing interest in developing a multimarker approach for identifying cardiovascular risk^37^,^38^,^39^. The novel markers may be potential targets that can be included in a multimarker panel for predicting CVD and identifying individuals at higher risk of MI, above that of traditional risk factors.

Our study has some limitations: We did not have accurate times to MI to our disposal, and despite exclusion of patients with clinical CVD, the group middle-aged relatively healthy individuals may constitute a heterogeneous study population with subclinical atherosclerosis at different stages.

In conclusion, elevated PTX3, elevated ficolin-2 and low MASP-3 concentrations were associated with increased risk of future MI in young to middle-aged relatively healthy Caucasians. Higher PTX3 and ficolin-2 concentrations may indicate higher complement activity, especially related to the lectin pathway. However, underlying mechanisms and potential use in a predictive setting merits further investigation. Nevertheless, our findings support the involvement of complement-dependent inflammation in the pathophysiology of cardiovascular disease.

## Materials and Methods

The present case-control study was generated from population data in the second wave of the Nord-Trøndelag Health (HUNT2) study, which was matched to data on incident acute MIs. The HUNT2 study was carried out in 1995–97 in the county of Nord-Trøndelag, Norway. This county is fairly representative for Norway as a whole. All inhabitants in the county of Nord-Trøndelag aged 13 and older were invited, and about 75,000 (70%) of those invited participated. Information was collected through comprehensive questionnaires and a clinical examination. A venous blood sample was drawn from attendants above 20 years. The inclusion process is described in detail elsewhere^40^. All methods were carried out in accordance with relevant guidelines and regulations. The study protocol was approved by the Regional Research Ethics Committee in Medicine, Central Norway (project numbers 157–1997 dated 06/11/1997 and 2009.1852 dated 11/20/2009) and the Data Inspectorate of Norway. Informed consent was obtained from all HUNT2 participants.

Data on MI hospitalization was collected from the two primary hospitals in the county of Nord-Trøndelag: Levanger Hospital and Namsos Hospital. From 1995 until 2000 data were registered retrospectively, and from 2001 registration has been done prospectively. The European Society of Cardiology/American College of Cardiology guidelines were used for verification of MI diagnosis. The criteria are elevated troponin T or troponin I together with at least one of the following: (1) symptoms consistent with myocardial infarction and/or (2) ECG changes with development of significant Q wave and/or (3) ECG changes consistent with ischemia (ST segment elevation or depression).

To be eligible for the present study, the participants of HUNT2 had to meet these criteria: available plasma and DNA as well as no previous self-reported cardiovascular disease. 57,133 persons met these criteria. By the end of 2008, 1,689 HUNT2 participants had been admitted with MI and the 370 youngest were selected as cases in the present study. A younger population was particularly selected because the investigated parameters may be less influenced by confounding factors than in an elderly population with comorbidities. Controls matched on age (±2 years) and gender were randomly selected, and all controls were at risk of MI at the time when their corresponding case experienced MI. The same study cohort was described for a previous study investigating polymorphisms in *MBL2* and ficolin genes in relation to the risk of MI^17^.

### Clinical variables

Clinical variables such as height, weight and blood pressure were registered as previously described^40^, and body mass index (BMI) was calculated. Hypertension was defined as systolic blood pressure ≥140 mmHg or as diastolic blood pressure ≥90 mmHg, or current use of antihypertensive medication. Information on other medication, such as statins was not available. Concentrations of glucose, creatinine and blood lipids were analysed by standard methods at the General Laboratory at Levanger Hospital. Hypercholesterolemia was defined as total cholesterol >6.2 mmol/L. Smoking was categorized into two groups: never/former or current smokers. The Framingham risk score^4^ was calculated using the variables in the HUNT2 database (age, HDL-cholesterol, total cholesterol, systolic blood pressure, antihypertensive treatment, smoking and diabetes). A positive family history for coronary heart disease was defined as MI before 60 years in a first-degree relative.

### Assays

Serum concentrations of ficolin-1, -2 and -3, PTX3, MASP-3 and MAP-1 were determined by sandwich ELISAs using specific in-house produced monoclonal antibodies as previously described^13^,^21^,^41^,^42^,^43^,^44^. All assays were optimized for automated analysis in the 384 well format on Biomek FX (Beckman Coulter, Fullerton, CA, USA).

### Statistical analyses

Due to non-normal distribution of several variables, the Mann-Whitney U test was used to evaluate differences in continuous variables. The Chi square test was used to compare categorical variables.

Linear regression with robust standard errors was used to analyse how clinical variables were associated with serum concentration of the biomarkers. The clinical variables included were: age, gender, smoking (yes/no), hypertension (yes/no), BMI, diabetes (yes/no), hypercholesterolemia (yes/no) and creatinine. Due to non-normality, either logarithmic transformation or Box-Cox transformation was performed, and extreme outliers were removed from the final models if they substantially altered the results.

Spearman correlation analysis was used to evaluate how the different biomarkers were related to each other. Associations between biomarker concentration and risk of MI were investigated using logistic regression. Biomarkers were divided into tertiles. Traditional risk factors included in the final models were sex, age, hypertension (yes/no), hypercholesterolemia (yes/no), smoking (never or previous/current), diabetes (yes/no) and BMI.

Finally, the improvement in predictive utility was explored with an analysis of the AUC, continuous net reclassification index and integrated discrimination index. Prediction of MI using the Framingham risk score alone was compared to a model combining the Framingham risk score with significant biomarkers from the multimarker analysis.

All tests were two-sided, and results are presented as medians or odds ratios with 95% confidence intervals. P-values below 0.05 were considered statistically significant. All analyses were performed with Stata/MP for Mac, version 11.2, (Stata Corp., College Station, Texas, USA) and R, version 3.2.2 (Foundation for Statistical Computing, Vienna, Austria).

## Additional Information

**How to cite this article:** Vengen, I. T. *et al*. Pentraxin 3, ficolin-2 and lectin pathway associated serine protease MASP-3 as early predictors of myocardial infarction - the HUNT2 study. *Sci. Rep.* **7**, 43045; doi: 10.1038/srep43045 (2017).

**Publisher's note:** Springer Nature remains neutral with regard to jurisdictional claims in published maps and institutional affiliations.

## Figures and Tables

**Table 1:** Patient characteristics.

	Cases	Controls	P-value
	(n = 366)	(n = 369)	
**Gender**, female/male	87/279	88/281	0.98
**Age**, years	48 (47–48)	48 (47–48)	0.94
**Age at MI,** years	53 (29–62)	—	—
**Body mass index**, kg/m^2^	27.4 (27.0–27.8)	26.5 (26.1–26.9)	0.004
**Hypertension**	193 (53%)	162 (44%)	0.02
- Systolic blood pressure, mmHg	140 (139–142)	136 (135–138)	< 0.001
- Diastolic blood pressure, mmHg	85 (84–87)	83 (82–84)	0.002
**Diabetes mellitus**	13 (4%)	4 (1%)	0.03
**Creatinine, μmol/L**	90 (89–91)	90 (89–92)	0.67
**Hypercholesterolemia**	242 (66%)	145 (39%)	<0.001
**Total cholesterol, mmol/L**	6.8 (6.7–6.9)	6.0 (5.9–6.2)	<0.001
**Triglycerides, mmol/L**	2.53 (2.35–2.70)	2.05 (1.91–2.19)	<0.001
**HDL cholesterol, mmol/L**	1.18 (1.14–1.23)	1.28 (1.25–1.32)	<0.001
**Smoking**			
- Former/never	133 (37%)	195 (56%)	<0.001
- Current	226 (63%)	155 (44%)	
**Framingham risk score**	13 (13– 14)	11 (11–12)	<0.001
Family history of MI*	100 (27%)	54 (15%)	<0.001

Continuous variables are presented as median (95% CI), categorical variables as n (%). *Myocardial infarction before 60 years in first-degree relatives. HDL, high-density lipoprotein; MI, myocardial infarction.

**Table 2:** Serum concentrations of the different biomarkers.

	Median concentration (±95% confidence interval)		P-value		Missing, %
	Cases	Controls	Unadjusted*	Adjusted	
Pentraxin-3, ng/ml	2.96 (2.75–3.17)	2.16 (1.99–2.35)	<0.001	<0.001	5.7%
Ficolin-1, μg/ml	0.44 (0.41–0.46)	0.47 (0.44–0.51)	0.31	0.63	0
Ficolin-2, μg/ml	5.07 (4.89–5.26)	4.57 (4.38–4.75)	<0.001	0.03	0
Ficolin-3, μg/ml	25.4 (24.3–26.5)	24.2 (23.1–25.4)	0.09	0.78	0.4%
MASP-3, μg/ml	4.50 (4.16–4.87)	4.98 (4.65–5.34)	0.033	0.03	2.0%
MAP-1, μg/ml	0.17 (0.16–0.18)	0.18 (0.17–0.19)	0.39	0.18	0

^*^Unadjusted analysis: P-values from Mann-Whitney U-test. Adjusted analysis: P-values from linear regression with robust standard errors with adjustments for age, sex, body mass index, creatinine, hypertension, hypercholesterolemia and smoking status. MASP-3, MBL/ficolin/collectin-associated serine protease-3: MAP-1, MBL/ficolin/collectin-associated protein-1.

**Table 3:** Logistic regression analysis.

	Unadjusted		Adjusted*	
	OR (95% CI)	P-value	OR (95% CI)	P-value
Pentraxin-3				
Tertile I	1.0		1.0	
Tertile II	1.72 (1.18–2.50)	**0.01**	1.65 (1.10–2.47)	**0.02**
Tertile III	2.87 (1.95–4.23)	**<0.001**	2.79 (1.83–4.24)	**<0.001**
Ficolin-1				
Tertile I	1.0		1.0	
Tertile II	1.02 (0.71–1.47)	0.9	1.10 (0.74–1.62)	0.64
Tertile III	0.79 (0.55–1.14)	0.21	0.77 (0.52–1.13)	0.18
Ficolin-2				
Tertile I	1.0		1.0	
Tertile II	1.64 (1.13–2.36)	**0.01**	1.36 (0.92–2.01)	0.13
Tertile III	2.00 (1.39–2.89)	**<0.001**	1.55 (1.04–2.30)	**0.03**
Ficolin-3				
Tertile I	1.0		1.0	
Tertile II	1.36 (0.95–1.96)	0.10	1.20 (0.81–1.77)	0.36
Tertile III	1.33 (0.92–1.91)	0.13	1.17 (0.79–1.73)	0.44
MASP-3				
Tertile I	1.0		1.0	
Tertile II	0.65 (0.45–0.93)	**0.02**	0.68 (0.46–1.01)	0.06
Tertile III	0.66 (0.46–0.95)	**0.02**	0.63 (0.43–0.94)	**0.02**
MAP-1				
Tertile I	1.0		1.0	
Tertile II	1.23 (0.85–1.77)	0.27	1.16 (0.78–1.71)	0.46
Tertile III	0.90 (0.63–1.30)	0.59	0.86 (0.59–1.27)	0.45

CI, confidence interval; MASP-3, MBL/ficolin/collectin-associated serine protease-3; MAP-1, MBL/ficolin/collectin-associated protein-1; OR, odds ratio.

^*^Adjusted for sex, age, hypertension (yes/no), hypercholesterolemia (yes/no), smoking (never or previous/current), diabetes (yes/no) and body mass index.

**Table 4:** Results from logistic regression including the three biomarkers that emerged as significant in primary analysis.

	Unadjusted		Adjusted*	
	OR (95% CI)	P-value	OR (95% CI)	P-value
PTX3				
Tertile I	1.0		1.0	
Tertile II	1.65 (1.12–2.44)	**0.011**	1.61 (1.07–2.44)	**0.023**
Tertile III	2.93 (1.96–4.37)	**<0.0005**	2.90 (1.89–4.47)	**<0.0005**
Ficolin-2				
Tertile I	1.0		1.0	
Tertile II	1.51 (1.03–2.24)	**0.037**	1.24 (0.82–1.88)	0.31
Tertile III	1.84 (1.24–2.73)	**0.003**	1.41 (0.92–2.16)	0.11
MASP-3				
Tertile I	1.0		1.0	
Tertile II	0.67 (0.45–1.0)	**0.049**	0.68 (0.45–1.05)	0.080
Tertile III	0.56 (0.38–0.82)	**0.003**	0.52 (0.34–0.79)	**0.002**

CI, confidence interval; MASP-3, MBL/ficolin/collectin-associated serine protease-3; MAP-1, MBL/ficolin/collectin-associated protein-1; OR, odds ratio.

^*^Adjusted for sex, age, hypertension (yes/no), hypercholesterolemia (yes/no), smoking (never or previous/current), diabetes (yes/no) and body mass index.
